# Indole-3-Acetic Acid as a Putative Selective AhR Modulator Counteracts Skatole-Induced Dual-Hit Toxicity in Colorectal Cancer Cells

**DOI:** 10.3390/toxins18020098

**Published:** 2026-02-14

**Authors:** Chihiro Takei, Hidehisa Shimizu

**Affiliations:** 1 Graduate School of Natural Science and Technology, Shimane University, 1060 Nishikawatsu-Cho, Matsue 690-8504, Shimane, Japan; 2 Faculty of Life and Environmental Sciences, Shimane University, 1060 Nishikawatsu-Cho, Matsue 690-8504, Shimane, Japan; 3 Estuary Research Center, Shimane University, 1060 Nishikawatsu-Cho, Matsue 690-8504, Shimane, Japan; 4 Interdisciplinary Center for Science Research, Shimane University, 1060 Nishikawatsu-Cho, Matsue 690-8504, Shimane, Japan; 5 Institute of Agricultural and Life Sciences, Academic Assembly, Shimane University, 1060 Nishikawatsu-Cho, Matsue 690-8504, Shimane, Japan

**Keywords:** skatole, indole-3-acetic acid, aryl hydrocarbon receptor (AhR), colorectal cancer, gut microbiota, uremic toxins, protein-bound solutes, MAPK, selective AhR modulator (SAhRM), dysbiosis

## Abstract

The rising incidence of colorectal cancer (CRC) in modernized societies is linked to diet-induced dysbiosis, characterized by a critical metabolic divergence: the depletion of protective indole-3-acetic acid (IAA) concurrent with the accumulation of toxic skatole (3-methylindole). However, the molecular mechanisms by which high concentrations of skatole drive malignancy—and whether IAA can counteract this toxicity—remain elusive. Here, we demonstrate that physiologically relevant concentrations of skatole (500 µM) significantly promote the proliferation of HCT-116 CRC cells through a “dual-hit” mechanism involving both aryl hydrocarbon receptor (AhR)-dependent genomic activity and AhR-independent activation of the ERK MAPK pathway. Notably, co-treatment with IAA (250 µM) effectively abrogated skatole-induced proliferation, restoring cell growth to baseline levels while sparing upstream MAPK phosphorylation. Mechanistic analysis indicates that IAA acts not merely as a competitor, but as a functional antagonist. Specifically, our findings suggest that IAA functions as a putative selective AhR modulator (SAhRM) that qualitatively reprograms AhR signaling. This modulation uncouples upstream MAPK phosphorylation from downstream cell cycle progression, effectively impeding the proliferative program even in the presence of skatole-induced stress. Furthermore, we propose a theoretical model of counter-balancing metabolic activation, hypothesizing that the oxidative environment associated with skatole metabolism may trigger the bioactivation of IAA into highly active anti-tumor derivatives. These findings suggest that restoring the gut IAA/skatole balance—either by targeting the bacterial enzyme indoleacetate decarboxylase (IAD) or via dietary resistant starch—may offer a promising precision nutrition strategy for CRC prevention.

## 1. Introduction

Colorectal cancer (CRC) stands as one of the leading causes of death in modern society [1,2,3]. The rapid rise in incidence, particularly in Asian countries including Japan, has escalated from a medical issue to a serious public health crisis [3]. Historically, Japan maintained a remarkably low global incidence of CRC, which was likely associated with a traditional diet rich in fermentable dietary fibers derived from brown rice, soybeans, and seaweeds. However, following the rapid economic growth after World War II, Japan’s dietary structure underwent a dramatic nutritional transition. The popularization of bread and dairy products, initiated by the School Lunch Act of 1954, and the dietary shift observed around 1985—where animal protein intake surpassed that of plant protein—fundamentally altered the Japanese intestinal environment [4]. Currently, the intake of red and processed meats among the Japanese population is on an upward trend [5], and it is widely acknowledged that this Westernization of diet correlates with the increase in inflammatory bowel diseases (IBD) and colorectal cancer risk in Japan [6,7,8,9].

**Figure 1:**
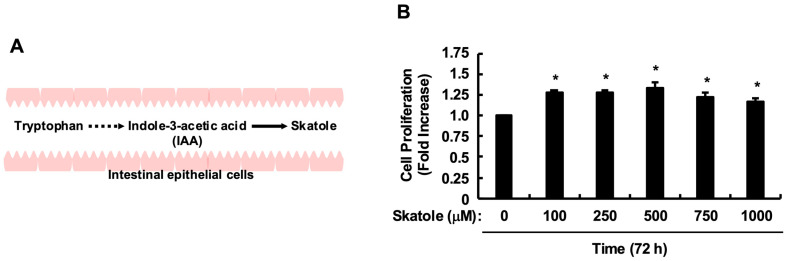
Effect of skatole on the proliferation of HCT-116 cells. (**A**) Schematic representation of tryptophan metabolism by gut microbiota. Tryptophan is converted into indole-3-acetic acid (IAA) or skatole (3-methylindole) depending on the metabolic pathway. Arrows indicate the metabolic conversion pathways. (**B**) Skatole induces cell proliferation. HCT-116 cells were treated with various concentrations of skatole (0–1000 µM) for 72 h. Cell proliferation was measured using the Cell Counting Kit-8. Data are expressed as fold increase relative to the vehicle control (0 µM). Values are mean ± SE of quadruplicates (*n* = 4 wells) from four independent experiments (*N* = 4 separate passages). * *p* < 0.05 vs. control (Dunnett’s test).

This dramatic dietary shift has fundamentally disrupted the metabolic balance of the essential amino acid tryptophan (Trp) by gut microbiota [10]. Unabsorbed Trp reaching the colon is converted into bioactive indole derivatives by various bacterial groups, but the metabolic pathway is heavily influenced by dietary content [10,11,12] (Figure 1A). Recent clinical studies highlight a critical finding: in healthy individuals, indole-3-acetic acid (IAA), which possesses anti-colorectal cancer activity and anti-inflammatory properties (e.g., via IL-35 induction [13]) that prevent tumorigenesis, is abundantly maintained. Specifically, IAA has been shown to inhibit tumorigenesis through IL-35 induction [13], ameliorate colitis via ERK signaling pathways [14], and maintain intestinal epithelial homeostasis through mucin sulfation [15]. This trend of IAA depletion is not limited to the local intestinal environment but has also been confirmed in the serum [16] and urine [17] of colorectal cancer patients, suggesting a systemic alteration in metabolic homeostasis. Conversely, in the feces of patients with a deteriorated intestinal environment, a metabolic divergence is observed where IAA levels significantly decrease while the concentration of the toxic metabolite skatole (3-methylindole) markedly increases [12]. Skatole is often discussed in the context of protein-bound uremic toxins that accumulate in chronic kidney disease (CKD) [18,19,20]; however, its local toxicity at the site of production—the gut—remains less understood. Skatole has been detected in the feces of patients with digestive disorders and colorectal cancer, reportedly reaching high concentrations of approximately 100 µg/g, which corresponds to roughly 1000 µM in molar concentration [11,12,21]. We hypothesize that this clinically observed profile... serves as a chemical driver of colorectal carcinogenesis, potentially through sustained perturbation of host AhR-dependent signaling pathways.

Furthermore, recent findings have revealed that CRC differs significantly in embryological origin and biological characteristics depending on the site of occurrence (proximal right side vs. distal left side). Right-sided colorectal cancer (RCC), in particular, has a poor prognosis and it is strongly suggested to involve the microbiota [6,22]. A pivotal role in the progression of RCC is played by the formation of bacterial biofilms specific to the right colon [22,23]. Within this biofilm microenvironment, it is plausible that specific IAD-expressing anaerobes, such as *Olsenella scatoligenes* and *Clostridium* species [24,25], catalyze the conversion of beneficial IAA into toxic skatole. Inside biofilms, diffusion of metabolites is restricted, suggesting that locally elevated concentrations of skatole may occur, continuously exposing host cell signal transduction systems. We propose that a Westernized diet promotes a synergistic pathological milieu where protective IAA is depleted while toxic skatole production is markedly increased. This metabolic shift from an ‘IAA-conserving’ to a ‘skatole-producing’ mode represents a critical turning point that may subvert the host’s Aryl Hydrocarbon Receptor (AhR) signaling from a protective mechanism into a driver of carcinogenesis. Elucidating the molecular consequences of this dysregulation is crucial for CRC prevention.

We have consistently investigated the impact of gut microbiota-derived tryptophan metabolites on colonic homeostasis and pathophysiology. Specifically, we have investigated complex inflammatory regulation mechanisms wherein skatole regulates intestinal epithelial cell functions via AhR and p38 pathways [26] and detailed the molecular mechanisms of skatole-induced inflammation [27,28]. We also reported that indoxyl sulfate acts as a toxic ligand promoting colorectal cancer cell proliferation via AhR/Akt and AhR/c-Myc pathways [29,30]. Conversely, regarding indole-3-acetic acid (IAA), we have demonstrated its anti-inflammatory and anti-proliferative effects via non-AhR pathways [31,32,33] in a cell-context dependent manner. Additionally, recent findings report that IAA reprograms systemic homeostasis and significantly ameliorates cachexia associated with IBD [34]. While our previous work established that toxic indoles function as potent AhR activators driving malignancy, the potential for IAA to qualitatively modulate AhR signaling itself remains a critical open question. Specifically, elucidating how a low-affinity ligand like IAA can functionally override the potent signaling driven by high concentrations of skatole constitutes the central mechanistic gap addressed in the present study. In this study, using HCT-116 cells which possess typical characteristics of RCC (MSI-H, *KRAS* mutation) [35], we aimed to elucidate the dual-hit proliferation mechanism induced by clinically relevant high concentrations of skatole. Furthermore, we aimed to evaluate whether IAA can act as a selective AhR modulator (SAhRM) by qualitatively modulating, rather than merely competing with, skatole-induced AhR signaling, thereby shifting cell fate from proliferation toward a tumor-suppressive response.

## 2. Results

### 2.1. Skatole Promotes Colorectal Cancer Cell Proliferation in an AhR-Dependent Manner

**Figure 2:**
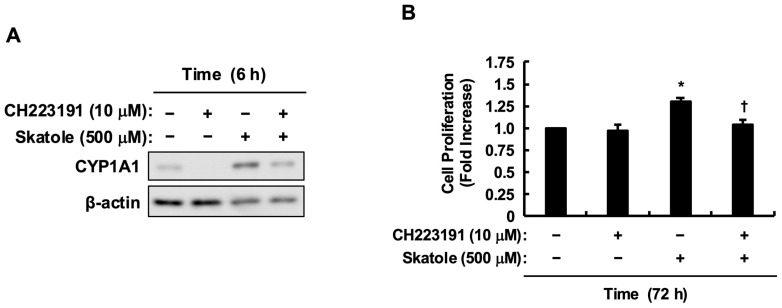
Skatole-induced proliferation is dependent on AhR activation. (**A**) Skatole induces *CYP1A1* protein expression via AhR. HCT-116 cells were pretreated with the AhR antagonist CH223191 (10 µM) for 1 h, followed by stimulation with skatole (500 µM) for 6 h. Whole cell lysates were analyzed by Western blotting for *CYP1A1* and β-actin (loading control). (**B**) AhR antagonism blocks skatole-induced cell proliferation. Cells were pretreated with CH223191 (10 µM) for 1 h and then stimulated with skatole (500 µM) for 72 h. Cell proliferation was assessed by Cell Counting Kit-8. Values are mean ± SE of quadruplicates (*n* = 4 wells) from four independent experiments (*N* = 4 separate passages). * *p* < 0.05 vs. control solution (DMSO), ^†^ *p* < 0.05 vs. skatole (Tukey–Kramer test).

First, we validated the physiological relevance of the skatole concentration used in this study. It has been reported that fecal skatole concentrations in patients with severe dysbiosis reach approximately 100 µg/g [11,12,21]. Assuming a fecal water content of 75%, 100 µg/g of feces corresponds to approximately 133 µg per mL of liquid phase. Given the molecular weight of skatole (131.17 g/mol), this equates to approximately 1015 µM. Importantly, recent studies highlight that right-sided colorectal tumors are often covered by dense bacterial biofilms [22,23], creating a local microenvironment where metabolite diffusion is restricted. Consequently, local concentrations can substantially exceed those found in the bulk fecal stream. Therefore, we established 1000 µM as a physiologically plausible maximum. Under these conditions, proliferation assays revealed that skatole significantly promoted the proliferation of HCT-116 cells across this clinically relevant range (100–1000 µM) (Figure 1B). Since the growth-promoting effect was maximal at 500 µM, we selected this concentration as a physiologically relevant estimate of localized epithelial exposure for subsequent mechanistic analyses. Next, to confirm whether skatole functions as a ligand for AhR, we examined changes in the expression level of the target gene *CYP1A1*. Pretreatment with an AhR antagonist (CH223191) markedly suppressed the upregulation of *CYP1A1* expression induced by skatole stimulation (Figure 2A). Furthermore, CH223191 significantly inhibited skatole-induced proliferation of HCT-116 cells, reducing the cell viability to levels comparable to the vehicle control (approximately 60–70% of the skatole-treated group) (Figure 2B). These results demonstrate that skatole at physiologically plausible concentrations drives the abnormal proliferation of colorectal cancer cells using AhR as a key mediator.

### 2.2. Activation of ERK Contributes to Skatole-Induced HCT-116 Cell Proliferation

**Figure 3:**
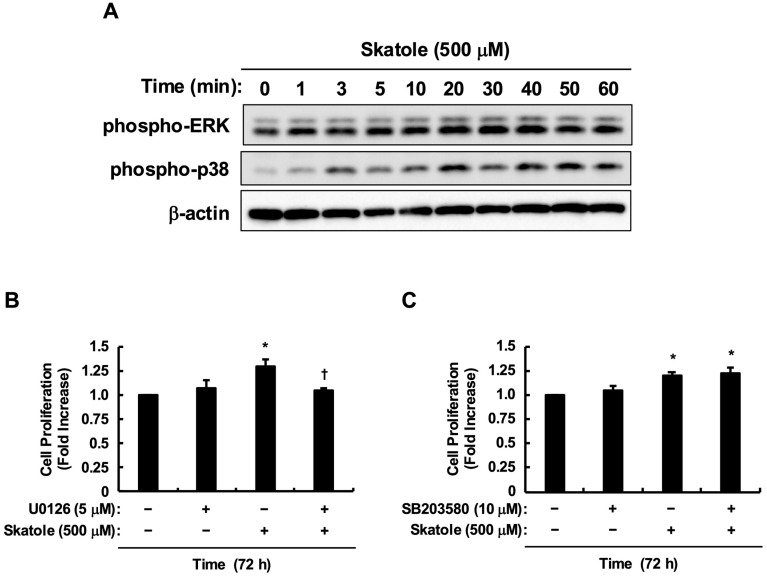
Activation of ERK, but not p38, drives skatole-induced proliferation. (**A**) Time course of MAPK phosphorylation. HCT-116 cells were stimulated with skatole (500 µM) for the indicated times (0–60 min). Phosphorylation levels of ERK1/2 and p38 were analyzed by Western blotting. β-Actin served as a loading control. (**B**) The MEK1/2 inhibitor (ERK pathway inhibitor) U0126 suppresses skatole-induced proliferation. Cells were pretreated with U0126 (5 µM) for 30 min prior to skatole (500 µM) stimulation for 72 h. (**C**) The p38 inhibitor SB203580 does not affect skatole-induced proliferation. Cells were pretreated with SB203580 (10 µM) for 30 min prior to skatole (500 µM) stimulation for 72 h. For (**B**,**C**), cell proliferation was determined by Cell Counting Kit-8. Values are mean ± SE of quadruplicates (*n* = 4 wells) from (**B**) six (*N* = 6 separate passages) and (**C**) five (*N* = 5 separate passages) independent experiments. * *p* < 0.05 vs. control solution (DMSO), ^†^ *p* < 0.05 vs. skatole. (Tukey–Kramer test).

Subsequently, we analyzed the intracellular signal transduction pathways induced by skatole. Given that ERK1/2 is a canonical driver of cell proliferation, and our previous findings identified p38 as a key downstream effector of skatole in Caco-2 cells [26], we focused our investigation on these two pathways. Addition of skatole (500 µM) resulted in rapid phosphorylation (activation) of both ERK1/2 and p38 (Figure 3A). To determine which MAPK pathway drives proliferation, we conducted experiments using specific inhibitors. The results showed that the ERK pathway inhibitor (U0126) significantly suppressed skatole-induced cell proliferation (Figure 3B). In contrast, the p38 inhibitor (SB203580) had no effect on proliferation, despite the observed phosphorylation of p38 (Figure 3C). Thus, these results indicate that while skatole activates multiple MAPK pathways, the activation of ERK specifically contributes to the induced proliferation of HCT-116 cells.

### 2.3. Skatole-Induced MAPK Activation Is Mediated via an AhR-Independent Pathway

**Figure 4:**
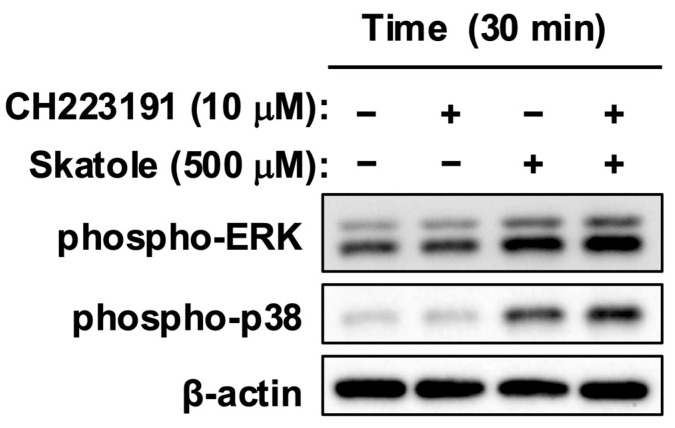
Skatole activates ERK and p38 via an AhR-independent pathway. HCT-116 cells were pretreated with the AhR antagonist CH223191 (10 µM) for 1 h, followed by stimulation with skatole (500 µM) for 30 min. Phosphorylation of ERK1/2 and p38 was analyzed by Western blotting. β-Actin served as a loading control. Note that AhR blockade did not inhibit skatole-induced MAPK activation, indicating a dual-hit mechanism where genomic (AhR) and nongenomic (MAPK) pathways operate independently.

Since skatole activated AhR and MAPKs (ERK and p38), we verified the possibility that AhR controls these kinase activities. As shown in Figure 4, pretreatment with the AhR antagonist (CH223191) did not alter the phosphorylation levels of either ERK or p38 induced by skatole. This indicates that AhR is not involved in skatole-mediated MAPK activation, suggesting the engagement of a parallel signal transduction pathway distinct from AhR. This finding implies that skatole drives proliferation signals by engaging an independent nongenomic pathway, separate from AhR-mediated genomic transcriptional activity. These results suggest that skatole toxicity involves a dual-hit mechanism that simultaneously mobilizes these two independent pathways.

### 2.4. IAA Abrogates Skatole-Induced Proliferation of HCT-116 Cells

**Figure 5:**
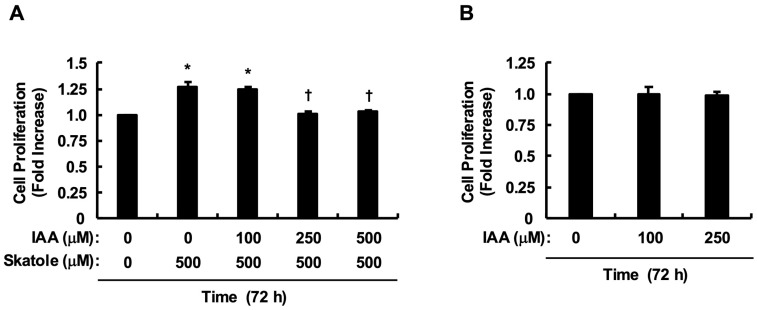
IAA counteracts skatole-induced proliferation without cytotoxicity. (**A**) IAA antagonizes the proliferative effect of skatole. HCT-116 cells were co-treated with skatole (500 µM) and increasing concentrations of IAA (0, 100, 250, 500 µM) for 72 h. IAA at 250 µM and 500 µM significantly abrogated skatole-induced proliferation. (**B**) IAA alone does not affect cell viability. Cells were treated with IAA (100, 250 µM) alone for 72 h. No significant cytotoxicity or proliferation was observed compared to the control. Values are mean ± SE of quadruplicates (*n* = 4 wells) from (**A**) four (*N* = 4 separate passages) and (**B**) three (*N* = 3 separate passages) independent experiments. * *p* < 0.05 vs. control solution (DMSO), ^†^ *p* < 0.05 vs. skatole. (Tukey–Kramer test).

We examined the effect of IAA, which is clinically depleted in colorectal cancer patients, on skatole-induced proliferation. Since skatole showed maximal growth promotion at 500 µM, we evaluated the antagonistic effect of IAA within the same concentration range. Notably, co-treatment with IAA dose-dependently suppressed skatole-induced proliferation. Notably, IAA at 250 µM was sufficient to effectively counteract the proliferation promotion, reducing cell numbers to control levels (*p* < 0.01 vs. skatole alone) (Figure 5A). Next, to rule out the possibility that this cancelation was due to nonspecific cytotoxicity, we examined the effect of IAA alone at the effective concentration range (0, 100, and 250 µM). As shown in Figure 5B, IAA alone did not affect cell proliferation, showing no significant difference compared to the control. These results support the notion that the rescue effect observed at 250 µM is due to the specific blockade of proliferation signals rather than cell death. Based on these findings, we selected 250 µM as the minimum effective and safe concentration for the subsequent mechanistic analyses.

### 2.5. IAA Modulates AhR Signaling and Blocks Proliferation While Maintaining ERK Activity

**Figure 6:**
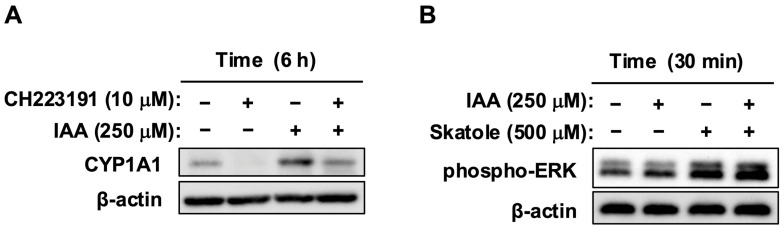
IAA modulates AhR signaling while preserving ERK activation (SAhRM-like activity). (**A**) IAA acts as an AhR ligand. HCT-116 cells were pretreated with CH223191 (10 µM) for 1 h and stimulated with IAA (250 µM) for 6 h. Whole cell lysates were analyzed by Western blotting for *CYP1A1* and β-actin (loading control). (**B**) IAA does not inhibit skatole-induced ERK phosphorylation. Cells were treated with skatole (500 µM) alone, IAA (250 µM) alone, or their combination for 30 min. Western blot analysis showed that ERK phosphorylation remained elevated in the co-treatment group, despite the suppression of proliferation (shown in Figure 5A), suggesting that IAA uncouples MAPK signaling from cell cycle progression. β-Actin served as a loading control.

We confirmed that IAA is also a ligand for AhR in HCT-116 cells, similar to skatole, through *CYP1A1* upregulation and its suppression by CH223191 (Figure 6A). Next, we examined whether IAA (250 µM) affects skatole-induced (500 µM) ERK activation. Interestingly, even while the proliferation-suppressive effect of IAA was being exerted, the activation of ERK—the proliferation accelerator induced by skatole—was maintained (Figure 6B). This paradoxical state suggests that IAA uncouples upstream MAPK survival signaling from the downstream cell cycle machinery. Typically, ERK activation drives proliferation; however, in the presence of IAA, this signal is uncoupled from cell division. These findings indicate that IAA does not merely turn off the signal source but rather induces a functional blockade at the receptor level, likely possessing properties of a SAhRM-like agent that induces a functional shift in AhR activity.

## 3. Discussion

### 3.1. Summary of Key Findings

**Figure 7:**
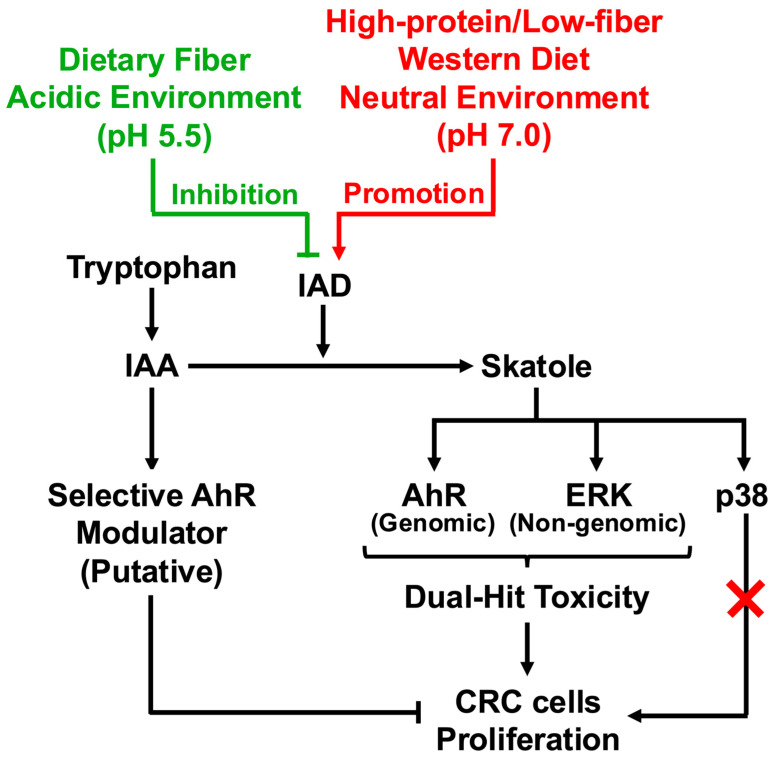
Proposed Graphical Model of Skatole-Induced Dual-Hit Toxicity and IAA-Mediated Rescue via SAhRM Mechanism. Under a high-protein/low-fiber Western diet, the colonic environment shifts to neutral pH (approx. pH 7.0), optimizing the activity of bacterial IAD. This accelerates the conversion of protective IAA into toxic skatole. Skatole drives CRC cell proliferation via a “dual-hit” mechanism: (1) AhR-dependent genomic signaling and (2) AhR-independent nongenomic ERK activation. While p38 is also phosphorylated, it does not contribute to the proliferative phenotype (indicated by a red cross). Conversely, dietary fiber intake restores an acidic environment (pH < 6.0), inhibiting IAD and conserving IAA. Conserved IAA acts as a putative SAhRM, functionally blocking the AhR-mediated proliferative program without inhibiting upstream ERK phosphorylation, thereby counteracting skatole toxicity. AhR, aryl hydrocarbon receptor; CRC, colorectal cancer cell; IAA, indole-3-acetic acid; IAD, indoleacetate decarboxylase; SAhRM, Selective AhR Modulator.

The present study elucidates the molecular mechanisms by which modern dietary changes drive bacterial metabolic switching toward skatole overproduction, and how resulting metabolite fluctuations shape host cell fate. We demonstrate that skatole, markedly elevated in CRC patient guts, potently drives HCT-116 cell proliferation through a dual-hit mechanism simultaneously engaging AhR-dependent genomic transcription and nongenomic MAPK (ERK1/2) activation. Critically, indole-3-acetic acid (IAA)—clinically depleted in CRC—appears to act as a functional selective antagonist. IAA qualitatively modulates AhR signaling at the receptor level while preserving skatole-induced MAPK activation, functioning as a putative selective AhR modulator (SAhRM) that potently counteracts skatole-driven proliferation. These findings suggest that bacterial IAD inhibition and IAA preservation via dietary intervention may offer a mechanistically rational strategy to disrupt CRC progression at the receptor level, as summarized in our proposed graphical model (Figure 7).

### 3.2. Mechanisms of Metabolic Dysregulation

The observation that skatole levels are pathologically elevated in CRC patients while IAA is depleted suggests a marked dysregulation of tryptophan metabolism homeostasis. We propose that the Westernized diet drives this “metabolic switch” through three converging mechanisms: enzymatic, genetic, and physical control. First, enzymatic regulation via pH plays a critical role. High dietary protein intake increases colonic ammonia production, elevating luminal pH [36]. Biochemical characterization of the skatole-producing enzyme, indoleacetate decarboxylase (IAD), reveals a strict pH optimum of approximately 7.5 (neutral to slightly alkaline), with sharply reduced activity in acidic conditions [24,37]. Consequently, the elevation of colonic pH typical of dysbiosis creates a highly favorable biochemical environment for IAD activity, accelerating the irreversible decarboxylation of the precursor IAA into toxic skatole. Second, genetic and ecological regulation exacerbates this process. In the absence of fermentable carbohydrates (dietary fiber), gut bacteria are released from carbon catabolite repression (CCR), triggering the expression of the tryptophanase gene (*tnaA*) to utilize amino acids as alternative energy sources [38]. Crucially, IAD expression is distinct from *tnaA* and is primarily governed by substrate induction; the accumulation of the precursor IAA paradoxically promotes the upregulation of IAD in specific anaerobes, such as *Olsenella scatoligenes* and *Clostridium* species [24,25,39]. This creates a pathological feed-forward loop where initial IAA accumulation fuels its own conversion to skatole. Third, physical retention amplifies metabolite accumulation. Low-fiber diets are associated with prolonged intestinal transit time (stasis) [40,41,42,43,44]. This stasis provides an extended fermentation window, favoring the proliferation of slow-growing anaerobes responsible for secondary metabolism and allowing skatole to accumulate to toxic concentrations within the lumen [45,46,47]. Collectively, these mechanisms provide a mechanistic basis for why the “High Skatole/Low IAA” metabolic profile is a hallmark of the Westernized gut environment.

### 3.3. Skatole-Induced Dual-Hit Proliferation Mechanism

AhR antagonism by CH223191 potently suppressed skatole-induced HCT-116 proliferation (Figure 2B), consistent with Karasová et al. [48], who demonstrated that AhR is an essential survival factor in these cells, regulating fatty acid synthase (FASN) to supply lipids for rapid membrane biogenesis. However, skatole-induced MAPK (ERK and p38) activation persisted despite complete AhR blockade (Figure 4). This indicates the existence of a parallel, AhR-independent MAPK activation pathway. While direct ROS quantification was not performed in this study, the metabolism of skatole by cytochrome P450 enzymes (e.g., *CYP1A1*, CYP2F1)—which are potently induced by skatole itself [49]—is well-established to generate reactive intermediates (3-methyleneindolenine) and ROS [12,49,50,51]. Critically, Schroyer et al. demonstrated that in HCT-116 cells, oxidative stress explicitly triggers ERK1/2 phosphorylation, which is requisite for driving a malignant phenotype [52]. This aligns with our finding that ERK inhibition significantly abolished skatole-induced proliferation (Figure 3B). Thus, our data suggest that skatole exerts a coordinated “dual-hit” control—simultaneously driving AhR-dependent transcriptional programs and ROS-mediated AhR-independent ERK hyperactivity [52]—which cooperatively fuels sustained CRC cell proliferation. Furthermore, we recently reported that indoxyl sulfate, another tryptophan-derived uremic toxin, exacerbates CRC progression by stabilizing the proto-oncogene c-Myc via an AhR/Akt-dependent pathway [30]. Given that ERK phosphorylation is a known stabilizer of c-Myc, it is highly plausible that skatole similarly exploits this “dual-hit” (AhR/ERK) axis to sustain high c-Myc levels, thereby fueling the rapid cell cycle progression characteristic of CRC. Verifying this downstream axis will be a critical objective of our future studies.

### 3.4. Cell-Specific Context of Skatole Toxicity

Remarkably, skatole at physiologically relevant concentrations (up to 1000 µM) drove robust proliferation in HCT-116 cells (Figure 1B), whereas we previously reported that it induces apoptosis in Caco-2 cells [26]. This dichotomous response likely reflects cell-specific metabolic vulnerabilities dictated by genetic background. Caco-2 cells (*KRAS* wild-type) express highly inducible *CYP1A1*, rapidly converting skatole to toxic 3-methyleneindolenine and generating overwhelming ROS [12,49], which exceeds their survival threshold. Conversely, HCT-116 cells (*KRAS* G13D mutant, MSI-H) [35], which represent a model of right-sided CRC, possess constitutively active survival signaling. We postulate that this oncogenic drive allows HCT-116 cells to tolerate skatole-derived oxidative stress and repurpose the concurrent AhR/ERK activation to fuel proliferation rather than cell death. This cell context-dependent fate switching provides mechanistic insight into the clinical paradox where skatole accumulation selectively promotes malignancy in specific CRC subsets, particularly within the biofilm-rich environment of the right colon [22,23].

### 3.5. IAA Functions as a Putative Selective AhR Modulator

Our data indicate that IAA does not merely compete with skatole for receptor binding but functions as a putative selective AhR modulator (SAhRM) that qualitatively reprograms downstream signaling [53,54,55]. The structural distinction between the flexible hydrophilic sidechain of IAA (-CH_2_COOH) and the hydrophobic methyl group of skatole (-CH_3_) likely induces thermodynamically divergent AhR conformations within the ligand-binding pocket [56,57]. Key evidence for this functional selectivity is the uncoupling of upstream signaling from downstream proliferation. As shown in Figure 6B, IAA completely abolished skatole-induced proliferation without inhibiting skatole-induced ERK phosphorylation. We postulate that IAA-bound AhR functionally overrides the proliferative ERK “accelerator”, potentially by acting as a dominant “molecular brake.” Mechanistically, ligand-activated AhR is known to directly induce the expression of the cyclin-dependent kinase inhibitor p27Kip1, leading to G1 cell cycle arrest [58]. Additionally, nongenomic modulation of other pathways [59] or interaction with the Hsp90 chaperone complex [60,61], and activation of the AhR E3 ubiquitin ligase function [62] may further contribute to this regulatory shift.

### 3.6. Metabolic Counter-Attack Hypothesis: Skatole-Driven Bioactivation of IAA

Beyond receptor competition, skatole metabolism by *CYP1A1* generates reactive intermediates (e.g., 3-methyleneindolenine) and ROS [12,49], establishing a pro-oxidant intracellular milieu. Crucially, the metabolic fates of skatole and IAA are diametrically opposed. Dong et al. recently demonstrated that unlike classical high-affinity ligands, IAA is not a substrate for *CYP1A1* despite inducing its expression [63]. Structural simulations reveal that IAA binds to an allosteric pocket distant from the catalytic heme center, thereby evading enzyme-dependent clearance [63]. Consequently, while skatole is rapidly metabolized, IAA persists intracellularly. This stability may allow IAA to undergo non-enzymatic ROS-mediated oxidation, yielding quinone-imine or dihydroxyindole derivatives with potentially enhanced bioactivity. Tintelnot et al. recently demonstrated that peroxidase-oxidized IAA intermediates suppress pancreatic malignancy [64], providing a direct precedent for this mechanism. It is tempting to speculate that the pathological ROS output driven by skatole might bioactivate its own metabolic antagonist IAA, turning the hostile microenvironment against the tumor cell.

### 3.7. Physiological Relevance of Metabolite Concentrations

A critical consideration for *in vitro* studies is the physiological relevance of the concentrations used. As summarized in Supplementary Table S1, reported fecal skatole concentrations in humans vary but can reach pathological levels in disease states. Notably, Karlin et al. reported skatole levels of approximately 100 µg/g feces in patients with digestive disorders [21]. Assuming a fecal water content of 75%, this corresponds to approximately 1000 µM in the aqueous phase. Furthermore, considering the restricted diffusion within the dense bacterial biofilms characteristic of right-sided CRC [22,23], epithelial cells are likely exposed to stable microgradients of skatole exceeding 1000 µM. Thus, the 500 µM concentration selected for our mechanistic experiments represents a conservative and highly relevant exposure level. Similarly, considering the stoichiometry of the reaction catalyzed by IAD (direct decarboxylation of IAA to skatole), the accumulation of skatole inherently implies an equimolar availability of its precursor, IAA. Therefore, suppressing IAD activity would theoretically allow the local IAA pool to recover to concentrations well exceeding the 250 µM used in our rescue experiments.

### 3.8. Limitations of the Present Study

While this study presents evidence for the “dual-hit” toxicity of skatole and the protective role of IAA, several limitations must be acknowledged. First, our functional assays primarily relied on the HCT-116 cell line. While this cell line models the specific context of right-sided, MSI-high, *KRAS*-mutated CRC, verifying these findings in patient-derived organoids would strengthen clinical translatability. However, it is noteworthy that human AhR has evolutionarily adapted to bind dietary indoles with higher specificity than murine AhR [65], suggesting that human cell models may provide more relevant mechanistic insights than rodent models. Second, although pharmacological inhibition using CH223191 effectively phenocopied AhR knockout effects [48], genetic ablation (CRISPR/Cas9) of AhR would provide definitive confirmation. Third, the designation of IAA as a “SAhRM” is currently based on functional outcomes (proliferation vs. signaling uncoupling); structural biology studies (e.g., X-ray crystallography) are required to elucidate the precise conformational changes induced by IAA binding. Finally, given the physicochemical distinction between lipophilic skatole and anionic IAA, their cellular uptake mechanisms likely differ. Therefore, elucidating the specific transporters responsible for IAA influx and examining how their expression levels influence its anti-proliferative efficacy remain important subjects for future investigation.

### 3.9. Clinical Translation: Precision Nutrition Strategy

Our findings suggest that the bacterial metabolic switch is a potential driver in the progression of CRC. High-fat, high-protein Western diets induce dysbiosis and disrupt protective tryptophan metabolic pathways [6,66]. A potential intervention strategy involves the restoration of physiological colonic pH. Daily intake of resistant starch promotes the production of short-chain fatty acids (SCFAs) and lowers colonic pH (<6.0) [39,67]. Since IAD belongs to the glycyl radical enzyme family and loses catalytic activity in acidic environments [24,37], luminal acidification could serve as a “metabolic brake,” inhibiting skatole production and conserving IAA [45]. Furthermore, microbial tryptophan metabolites have been shown to modulate intestinal immunity [68,69,70], engaging AhR to balance mucosal reactivity [69,71]. Thus, targeting bacterial IAD via precision nutrition may shift colonic metabolism from skatole-generation to IAA-conservation, potentially restoring the protective AhR axis.

## 4. Conclusions

The present study indicates that skatole drives HCT-116 cell proliferation via a dual-hit mechanism involving AhR-dependent genomic signaling and AhR-independent ERK activation. IAA counteracts this toxicity, functioning as a putative SAhRM that qualitatively reprograms receptor output without inhibiting upstream MAPK signaling. These findings provide a molecular basis for understanding how diet-induced dysbiosis contributes to CRC progression and suggest that restoring the IAA/skatole balance may offer a potential preventative strategy.

## 5. Materials and Methods

### 5.1. Materials

Various reagents and antibodies were obtained from suppliers for the present study. 3-Methylindole (Skatole; >98.0% GC) and 3-Indoleacetic Acid (>98.0%(T)) were purchased from Tokyo Chemical Industry (Tokyo, Japan). Cayman Chemical (Ann Arbor, MI, USA) provided the aryl hydrocarbon receptor (AhR) antagonist CH223191. The MEK1/2 (ERK pathway) inhibitor U0126, the p38 inhibitor SB203580, Dulbecco’s modified Eagle’s medium (DMEM), penicillin-streptomycin solution, and dimethyl sulfoxide (DMSO) were obtained from Wako Pure Chemical Industries (Osaka, Japan). Anti-β-actin antibody (C4) was supplied by Santa Cruz Biotechnology, Inc. (Dallas, TX, USA). Anti-phospho-p44/42 MAPK (ERK1/2) (Thr202/Tyr204) and anti-phospho-p38 (Thr180/Tyr182) antibodies were supplied by Cell Signaling Technology, Inc. (Danvers, MA, USA). Anti-*CYP1A1* antibody was obtained from Proteintech Group Inc. (Chicago, IL, USA). Peroxidase AffiniPure Goat Anti-Rabbit IgG (H + L) and Anti-Mouse IgG (H + L) antibodies were acquired from Jackson ImmunoResearch Laboratories, Inc. (West Grove, PA, USA). Nacalai Tesque (Kyoto, Japan) supplied Protease Inhibitor Cocktail (EDTA-free), Phosphatase Inhibitor Cocktail, and the chemiluminescent reagent Chemi-Lumi One L/Super. Fetal Bovine Serum (FBS) was procured from Biowest (Nuaillé, France).

### 5.2. Cell Culture

As this study utilized established cell lines *in vitro*, clinical inclusion/exclusion criteria are not applicable. The human colorectal cancer cell line HCT-116 was provided by the RIKEN BioResource Center (Tsukuba, Japan). Cells were maintained in DMEM supplemented with 10% FBS and 1% penicillin-streptomycin at 37 °C in a 5% CO2 atmosphere. Twenty-four hours prior to the start of experiments, cells were switched to serum-free DMEM to induce starvation and stabilize the signal baseline. DMSO was used as the solvent for all compounds (skatole, IAA, CH223191, inhibitors), and the final DMSO concentration in the medium was unified at 0.1% (*v*/*v*). The control group received the same concentration of DMSO (vehicle control).

### 5.3. Quantitation of Cell Proliferation

The proliferative capacity of HCT-116 cells was evaluated using the Cell Counting Kit-8 (Dojindo, Kumamoto, Japan). Cells were seeded in 96-well plates and starved, then treated with various concentrations of skatole (0–1000 µM) or IAA (0, 100, 250, 500 µM) alone or in combination for 72 h. When using the antagonist CH223191 (10 μM) or each signaling inhibitor, pretreatment was performed 30–60 min before ligand stimulation. After treatment, CCK-8 solution was added, and absorbance at 450 nm was measured using a microplate reader. Results were calculated as relative absorbance (% of control) or cell proliferation rate (fold increase) relative to the vehicle control.

### 5.4. Immunoblotting

Immunoblotting was performed to analyze signal transduction and target proteins. Stimulated HCT-116 cells were lysed on ice using a buffer as described in previous studies [29,30]. Proteins were separated using sodium dodecyl sulfate-polyacrylamide gel electrophoresis and transferred onto Immobilon-P polyvinylidene fluoride membranes (Millipore Inc., Bedford, MA, USA). After blocking, membranes were incubated with primary antibodies (anti-phospho-p44/42 MAPK (ERK1/2) (Thr202/Tyr204), anti-*CYP1A1*, and anti-β-actin at 1:5000 dilution; anti-phospho-p38 (Thr180/Tyr182) at 1:1000 dilution). After washing, membranes were incubated with the corresponding HRP-conjugated secondary antibodies (Goat Anti-Rabbit IgG or Anti-Mouse IgG; 1:5000 dilution). Target proteins were visualized using the Chemi-Lumi One system. Images were captured using an ImageQuant LAS 4010 (GE Healthcare). Membranes were stripped and reprobed to detect multiple proteins (*CYP1A1*, phospho-ERK, phospho-p38, and β-actin) on the same blot.

### 5.5. Statistical Analysis

All data are presented as mean ± standard error (SE). Data represent the mean of quadruplicates (*n* = 4 wells) from at least three independent experiments (*N* = 3–6 separate passages, as indicated in each figure legend). Statistical significance was evaluated using one-way analysis of variance (ANOVA), followed by post hoc tests. Specifically, Dunnett’s test was used for Figure 1, while the Tukey–Kramer test was applied for Figure 2A, Figure 3B,C and Figure 5A,B. All statistical analyses were performed using Microsoft Excel and Statcel 4 software (OMS Publishing, Saitama, Japan), with *p* < 0.05 considered statistically significant.

## Data Availability

The original contributions presented in this study are included in this article. Further inquiries can be directed to the corresponding author.

## Supplementary Materials

The following supporting information can be downloaded at: https://www.mdpi.com/article/10.3390/toxins18020098/s1, Table S1: Reported concentrations of skatole in human feces.

## References

[1] Siegel R.L., Giaquinto A.N., Jemal A. (2024). Cancer statistics, 2024. CA Cancer J. Clin..

[2] Bray F., Laversanne M., Sung H., Ferlay J., Siegel R.L., Soerjomataram I., Jemal A. (2024). Global cancer statistics 2022: GLOBOCAN estimates of incidence and mortality worldwide for 36 cancers in 185 countries. CA Cancer J. Clin..

[3] Xi Y., Xu P. (2021). Global colorectal cancer burden in 2020 and projections to 2040. Transl. Oncol..

[4] Otsuka R., Yatsuya H., Tamakoshi K., Imai T., Nishio N., Suzuki S., Chikaraishi Y., Kato Y., Ando M., Murata C. (2014). Descriptive epidemiological study of food intake among Japanese adults: Analyses by age, time and birth cohort model. BMC Public Health.

[5] Sasaki K., Motoyama M., Watanabe G., Nakajima I. (2022). Meat consumption and consumer attitudes in Japan: An overview. Meat Sci..

[6] Brennan C.A., Garrett W.S. (2016). Gut Microbiota, Inflammation, and Colorectal Cancer. Annu. Rev. Microbiol..

[7] Zhang Y., Yu Y., Jiang Z., Yu J., Zhang Z., An Z., Du Y., Mao Y., Hu L., Tang X. (2025). The impact of red meat and processed meat consumption on the risk of development and relapse of ulcerative colitis: A systematic review and dose-response meta-analysis. Front. Nutr..

[8] Chen H., Fu T., Dan L., Chen X., Sun Y., Chen J., Wang X., Hesketh T. (2022). Meat consumption and all-cause mortality in 5763 patients with inflammatory bowel disease: A retrospective cohort study. EClinicalMedicine.

[9] Takachi R., Tsubono Y., Baba K., Inoue M., Sasazuki S., Iwasaki M., Tsugane S. (2011). Red meat intake may increase the risk of colon cancer in Japanese, a population with relatively low red meat consumption. Asia Pac. J. Clin. Nutr..

[10] Roager H.M., Licht T.R. (2018). Microbial tryptophan catabolites in health and disease. Nat. Commun..

[11] Yokoyama M.T., Carlson J.R. (1979). Microbial metabolites of tryptophan in the intestinal tract with special reference to skatole. Am. J. Clin. Nutr..

[12] Zgarbová E., Vrzal R. (2023). Skatole: A thin red line between its benefits and toxicity. Biochimie.

[13] Wang J., Hao Y., Yang Y., Zhang Y., Xu C., Yang R. (2025). Gut microbiota derived indole-3-acetic acid ameliorates precancerous inflammatory intestinal milieu to inhibit tumorigenesis through IL-35. J. Immunother. Cancer.

[14] Qu X., Song Y., Li Q., Xu Q., Li Y., Zhang H., Cheng X., Mackay C.R., Wang Q., Liu W. (2024). Indole-3-acetic acid ameliorates dextran sulfate sodium-induced colitis via the ERK signaling pathway. Arch. Pharm. Res..

[15] Li M., Ding Y., Wei J., Dong Y., Wang J., Dai X., Yan J., Chu F., Zhang K., Meng F. (2024). Gut microbiota metabolite indole-3-acetic acid maintains intestinal epithelial homeostasis through mucin sulfation. Gut Microbes.

[16] Qiu Y., Cai G., Su M., Chen T., Zheng X., Xu Y., Ni Y., Zhao A., Xu L.X., Cai S. (2009). Serum metabolite profiling of human colorectal cancer using GC-TOFMS and UPLC-QTOFMS. J. Proteome Res..

[17] Cheng Y., Xie G., Chen T., Qiu Y., Zou X., Zheng M., Tan B., Feng B., Dong T., He P. (2012). Distinct urinary metabolic profile of human colorectal cancer. J. Proteome Res..

[18] Madella A.M., Van Bergenhenegouwen J., Garssen J., Masereeuw R., Overbeek S.A. (2022). Microbial-Derived Tryptophan Catabolites, Kidney Disease and Gut Inflammation. Toxins.

[19] Xie H., Yang N., Yu C., Lu L. (2024). Uremic toxins mediate kidney diseases: The role of aryl hydrocarbon receptor. Cell. Mol. Biol. Lett..

[20] Graboski A.L., Redinbo M.R. (2020). Gut-Derived Protein-Bound Uremic Toxins. Toxins.

[21] Karlin D.A., Mastromarino A.J., Jones R.D., Stroehlein J.R., Lorentz O. (1985). Fecal skatole and indole and breath methane and hydrogen in patients with large bowel polyps or cancer. J. Cancer Res. Clin. Oncol..

[22] Dejea C.M., Wick E.C., Hechenbleikner E.M., White J.R., Mark Welch J.L., Rossetti B.J., Sears C.L. (2014). Microbiota organization is a distinct feature of proximal colorectal cancer. Proc. Natl. Acad. Sci. USA.

[23] Bullman S., Pedamallu C.S., Sicinska E., Clancy T.E., Zhang X., Cai D., Neuberg D., Huang K., Guevara F., Nelson T. (2017). Analysis of Fusobacterium persistence and antibiotic response in colorectal cancer. Science.

[24] Liu D., Wei Y., Liu X., Zhou Y., Jiang L., Yin J., Wang F., Hu Y., Nanjaraj Urs A.N., Liu Y. (2018). Indoleacetate decarboxylase is a glycyl radical enzyme catalysing the formation of malodorant skatole. Nat. Commun..

[25] Whitehead T.R., Price N.P., Drake H.L., Cotta M.A. (2008). Catabolic pathway for the production of skatole and indoleacetic acid by the acetogen Clostridium drakei, Clostridium scatologenes, and swine manure. Appl. Environ. Microbiol..

[26] Kurata K., Kawahara H., Nishimura K., Jisaka M., Yokota K., Shimizu H. (2019). Skatole regulates intestinal epithelial cellular functions through activating aryl hydrocarbon receptors and p38. Biochem. Biophys. Res. Commun..

[27] Kurata K., Ishii K., Koto Y., Naito K., Yuasa K., Shimizu H. (2023). Skatole-induced p38 and JNK activation coordinately upregulates, whereas AhR activation partially attenuates TNFα expression in intestinal epithelial cells. Biosci. Biotechnol. Biochem..

[28] Ishii K., Naito K., Tanaka D., Koto Y., Kurata K., Shimizu H. (2024). Molecular Mechanisms of Skatole-Induced Inflammatory Responses in Intestinal Epithelial Caco-2 Cells: Implications for Colorectal Cancer and Inflammatory Bowel Disease. Cells.

[29] Ichisaka Y., Yano S., Nishimura K., Niwa T., Shimizu H. (2024). Indoxyl sulfate contributes to colorectal cancer cell proliferation and increased EGFR expression by activating AhR and Akt. Biomed. Res..

[30] Ichisaka Y., Takei C., Naito K., Higa M., Yano S., Niwa T., Shimizu H. (2025). The Role of Indoxyl Sulfate in Exacerbating Colorectal Cancer During Chronic Kidney Disease Progression: Insights into the Akt/β-Catenin/c-Myc and AhR/c-Myc Pathways in HCT-116 Colorectal Cancer Cells. Toxins.

[31] Tomii A., Higa M., Naito K., Kurata K., Kobayashi J., Takei C., Yuasa K., Koto Y., Shimizu H. (2023). Activation of the TLR4-JNK but not the TLR4-ERK pathway induced by indole-3-acetic acid exerts anti-proliferative effects on Caco-2 cells. Biosci. Biotechnol. Biochem..

[32] Chowdhury M.M.I., Kurata K., Yuasa K., Koto Y., Nishimura K., Shimizu H. (2021). Suppression of TNFα expression induced by indole-3-acetic acid is not mediated by AhR activation in Caco-2 cells. Biosci. Biotechnol. Biochem..

[33] Chowdhury M.M.I., Tomii A., Ishii K., Tahara M., Hitsuda Y., Koto Y., Kurata K., Yuasa K., Nishimura K., Shimizu H. (2021). TLR4 may be a novel indole-3-acetic acid receptor that is implicated in the regulation of CYP1A1 and TNFα expression depending on the culture stage of Caco-2 cells. Biosci. Biotechnol. Biochem..

[34] Tomii A., Takei C., Yoshikiyo K., Shimizu H. (2025). The Gut Microbial Metabolite Indole-3-Acetic Acid Reprograms Systemic Homeostasis and Ameliorates IBD-Associated Cachexia Independent of Food Intake. Int. J. Mol. Sci..

[35] Ahmed D., Eide P.W., Eilertsen I.A., Danielsen S.A., Eknaes M., Hektoen M., Lind G.E., Lothe R.A. (2013). Epigenetic and genetic features of 24 colon cancer cell lines. Oncogenesis.

[36] Yao C.K., Muir J.G., Gibson P.R. (2016). Review article: Insights into colonic protein fermentation, its modulation and potential health implications. Aliment. Pharmacol. Ther..

[37] Fu B., Levin B.J., Balskus E.P. (2022). Mechanistic Studies of a Skatole-Forming Glycyl Radical Enzyme Suggest Reaction Initiation via Hydrogen Atom Transfer. J. Am. Chem. Soc..

[38] Sinha A.K., Laursen M.F., Brinck J.E., Limborg M.T., Licht T.R., Roager H.M. (2024). Dietary fibre directs microbial tryptophan metabolism via metabolic interactions in the gut microbiota. Nat. Microbiol..

[39] Huang Z., Zhang X., Wells J.M., Fogliano V. (2023). Impact of High-Fiber or High-Protein Diet on the Capacity of Human Gut Microbiota To Produce Tryptophan Catabolites. J. Agric. Food Chem..

[40] Cummings J.H., Bingham S.A., Heaton K.W., Eastwood M.A. (1992). Fecal weight, colon cancer risk, and dietary intake. Gastroenterology.

[41] O’Keefe S.J., Li S.C., Lahti L., Ou J., Carbonero F., Mohammed K., Posma J.M., Kinross J., Khanna S., Nicholson J. (2015). Fat, fibre and cancer risk in African Americans and rural Africans. Nat. Commun..

[42] Anitha M., Vijay-Kumar M., Sitaraman S.V., Gewirtz A.T., Srinivasan S. (2016). Intestinal Dysbiosis Contributes to the Delayed Gastrointestinal Transit in High-Fat Diet Fed Mice. Cell. Mol. Gastroenterol. Hepatol..

[43] Reichardt F., Chassaing B., Nezami B.G., Li G., Tabatabai M.A., Mwangi S.M., Gewirtz A.T., Srinivasan S. (2017). Western diet induces colonic nitrergic myenteric neuropathy and dysmotility in mice via saturated fatty acid-induced oxidative stress. Am. J. Physiol. Gastrointest. Liver Physiol..

[44] Mushref M.A., Srinivasan S. (2013). Effect of high fat-diet and obesity on gastrointestinal motility. Ann. Transl. Med..

[45] Jensen B.B., Hansen L.L., Mikkelsen L.L. (1995). Microbial production of skatole in the hind gut of pigs given different diets and its relation to skatole deposition in backfat. Anim. Sci..

[46] Harvey R.F., Pomare E.W., Heaton K.W. (1973). Effects of increased dietary fibre on intestinal transit. Lancet.

[47] Hawe S.M., Walker N., Moss B.W. (1992). The effects of dietary fibre, lactose and antibiotic on the levels of skatole and indole in faeces and subcutaneous fat in growing pigs. Anim. Sci..

[48] Karasová M., Procházková J., Tylichová Z., Fedr R., Ciganek M., Machala M., Dvořák Z., Vyhlídalová B., Zůvalová I., Ehrmann J. (2022). Inhibition of Aryl Hydrocarbon Receptor (AhR) Expression Disrupts Cell Proliferation and Alters Energy Metabolism and Fatty Acid Synthesis in Colon Cancer Cells. Cancers.

[49] Rasmussen M.K., Balaguer P., Ekstrand B., Daujat-Chavanieu M., Gerbal-Chaloin S. (2016). Skatole (3-Methylindole) Is a Partial Aryl Hydrocarbon Receptor Agonist and Induces CYP1A1/2 and CYP1B1 Expression in Primary Human Hepatocytes. PLoS ONE.

[50] Weems J.M., Yost G.S. (2010). 3-Methylindole metabolites induce lung CYP1A1 and CYP2F1 enzymes by AhR and non-AhR mechanisms, respectively. Chem. Res. Toxicol..

[51] Ruangyuttikarn W., Appleton M.L., Yost G.S. (1991). Metabolism of 3-methylindole in human tissues. Drug Metab. Dispos..

[52] Schroyer A.L., Fymes X., Chadee D.N. (2018). MLK3 phosphorylation by ERK1/2 is required for oxidative stress-induced invasion of colorectal cancer cells. Oncogene.

[53] Xie G., Peng Z., Raufman J.P. (2012). Src-mediated aryl hydrocarbon and epidermal growth factor receptor cross talk stimulates colon cancer cell proliferation. Am. J. Physiol. Gastrointest. Liver Physiol..

[54] Vyhlídalová B., Krasulová K., Pečinková P., Marcalíková A., Vrzal R., Zemánková L., Vančo J., Trávníček Z., Vondráček J., Karasová M. (2020). Gut Microbial Catabolites of Tryptophan Are Ligands and Agonists of the Aryl Hydrocarbon Receptor: A Detailed Characterization. Int. J. Mol. Sci..

[55] Safe S., Jin U.H., Park H., Chapkin R.S., Jayaraman A. (2020). Aryl Hydrocarbon Receptor (AHR) Ligands as Selective AHR Modulators (SAhRMs). Int. J. Mol. Sci..

[56] Jin U.H., Lee S.O., Sridharan G., Lee K., Davidson L.A., Jayaraman A., Chapkin R.S., Alaniz R., Safe S. (2014). Microbiome-derived tryptophan metabolites and their aryl hydrocarbon receptor-dependent agonist and antagonist activities. Mol. Pharmacol..

[57] Denison M.S., Soshilov A.A., He G., DeGroot D.E., Zhao B. (2011). Exactly the same but different: Promiscuity and diversity in the molecular mechanisms of action of the aryl hydrocarbon (dioxin) receptor. Toxicol. Sci..

[58] Kolluri S.K., Weiss C., Koff A., Göttlicher M. (1999). p27(Kip1) induction and inhibition of proliferation by the intracellular Ah receptor in developing thymus and hepatoma cells. Genes Dev..

[59] Pallotta M.T., Fallarino F., Matino D., Macchiarulo A., Orabona C. (2014). AhR-mediated, non-genomic modulation of IDO1 function. Front. Immunol..

[60] Soshilov A., Denison M.S. (2011). Ligand displaces heat shock protein 90 from overlapping binding sites within the aryl hydrocarbon receptor ligand-binding domain. J. Biol. Chem..

[61] Genest O., Wickner S., Doyle S.M. (2019). Hsp90 and Hsp70 chaperones: Collaborators in protein remodeling. J. Biol. Chem..

[62] Luecke-Johansson S., Gralla M., Rundqvist H., Ho J.C., Johnson R.S., Gradin K., Poellinger L. (2017). A Molecular Mechanism To Switch the Aryl Hydrocarbon Receptor from a Transcription Factor to an E3 Ubiquitin Ligase. Mol. Cell. Biol..

[63] Dong H., Murray I.A., Annalora A.J., Zhang Q., Perdew G.H. (2023). Endogenous Tryptophan-Derived Ah Receptor Ligands are Dissociated from CYP1A1/1B1-Dependent Negative-Feedback. Int. J. Tryptophan Res..

[64] Tintelnot J., Xu Y., Lesker T.R., Schönlein M., Konczalla L., Giannou A.D., Pelczar P., Kylies D., Puelles V.G., Bielecka A.A. (2023). Microbiota-derived 3-IAA influences chemotherapy efficacy in pancreatic cancer. Nature.

[65] Hubbard T.D., Murray I.A., Bisson W.H., Lahoti T.S., Gowda K., Amin S.G., Patterson A.D., Perdew G.H. (2015). Adaptation of the human aryl hydrocarbon receptor to sense microbiota-derived indoles. Sci. Rep..

[66] Shi J., Zhao D., Zhao F., Wang C., Zamaratskaia G., Li C. (2021). Chicken-eaters and pork-eaters have different gut microbiota and tryptophan metabolites. Sci. Rep..

[67] Le Leu R.K., Brown I.L., Hu Y., Morita T., Esterman A., Young G.P. (2007). Effect of dietary resistant starch and protein on colonic fermentation and intestinal tumourigenesis in rats. Carcinogenesis.

[68] Krishnan S., Ding Y., Saedi N., Choi M., Sridharan G.V., Sherr D.H., Yarmush M.L., Alaniz R.C., Jayaraman A., Lee K. (2018). Gut Microbiota-Derived Tryptophan Metabolites Modulate Inflammatory Response in Hepatocytes and Macrophages. Cell Rep..

[69] Zelante T., Iannitti R.G., Cunha C., De Luca A., Giovannini G., Pieraccini G., Zecchi R., D’Angelo C., Massi-Benedetti C., Fallarino F. (2013). Tryptophan catabolites from microbiota engage aryl hydrocarbon receptor and balance mucosal reactivity via interleukin-22. Immunity.

[70] Gao J., Xu K., Liu H., Liu G., Bai M., Peng C., Li T., Yin Y. (2018). Impact of the Gut Microbiota on Intestinal Immunity Mediated by Tryptophan Metabolism. Front. Cell. Infect. Microbiol..

[71] Lamas B., Richard M.L., Leducq V., Pham H.P., Michel M.L., Da Costa G., Bridonneau C., Jegou S., Hoffmann T.W., Natividad J.M. (2016). CARD9 impacts colitis by altering gut microbiota metabolism of tryptophan into aryl hydrocarbon receptor ligands. Nat. Med..

