# Supplementary material for: Indole-3-Acetic Acid as a Putative Selective AhR Modulator Counteracts Skatole-Induced Dual-Hit Toxicity in Colorectal Cancer Cells
Source: Toxins (Basel). 2026 Feb 14;18(2):98. doi: 10.3390/toxins18020098 (PMC12944917; doi:10.3390/toxins18020098)
Supplement: Supplementary file 1 [file toxins-18-00098-s001.zip › toxins-4122024-supplementary.pdf]

Supplementary Table S1. Reported concentrations of skatole in human feces.

Study (Reference)	Subject Group	Reported Concentration	*Estimated Molar Concentration**
Yokoyama et al. (1979) [11]	Healthy adults	Range: 1.2 – 107 μg/g dry wt	~ 2 – 270 μM
Zgarbová et al. (2023) [12]	Review (Pathological)	General Ref: ~ About 100 μg/g	~ About 1000 μM
Karlin et al. (1985) [21]	Patients with gastrointestinal disease	Max: ~ About 100 μg/g wet wt	~ About 1000 μM

*Estimated molar concentrations in the liquid phase assume 75% fecal water content for wet weight values, or are derived from dry weight assuming standard hydration factors.
